# Underlap suturing: a safe closure technique for full-thickness defects using endoscopic hand suturing

**DOI:** 10.1055/a-2970-7683

**Published:** 2026-10-12

**Authors:** Yumiko Ishikawa, Kentaro Hara, Haruhiko Cho, Toshiro Iizuka, Osamu Goto

**Affiliations:** 1 Department of Gastroenterology 118084 Tokyo Metropolitan Cancer and Infectious Diseases Center Komagome HospitalTokyoJapan; 2 Department of Gastric Surgery 118084 Tokyo Metropolitan Cancer and Infectious Diseases Center Komagome HospitalTokyoJapan; 3 Department of Endoscopy 118084 Tokyo Metropolitan Cancer and Infectious Diseases Center Komagome HospitalTokyoJapan

Endoscopic hand suturing (EHS) is a reliable method of closing the surgical defect caused during endoscopic full-thickness resection (EFTR) 1​ 2​ 3​ ​4​ and is preferrable to full-thickness closure, which carries the risk of injury to adjacent organs caused by the needle. 5​ We herein describe the “underlap suturing” method, which we developed as a safe EHS technique for use in EFTR ( Fig. 1 , Video 1) .

**Fig. 1:**
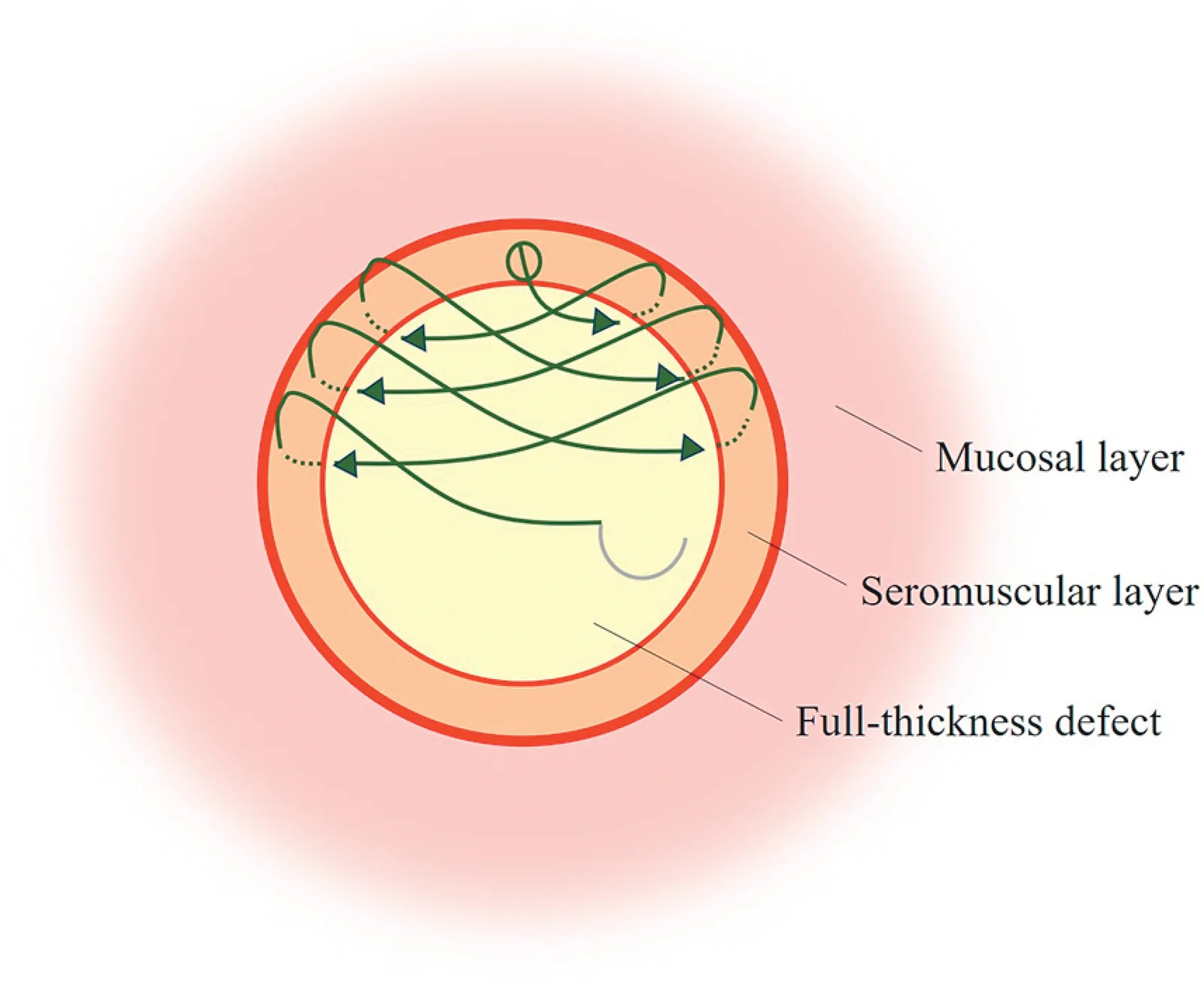
Schematic illustration of the underlap suturing technique. The needle is passed alternately through the left and right edges of the full-thickness defect from the peritoneal side toward the gastric lumen.

**Video 1** After removal of the gastric subepithelial lesion, the surgical defect was completely closed using the underlap technique, which enabled safe and reliable closure of the seromuscular layer.


A female patient in her 70’s underwent EFTR for a 3-cm gastrointestinal stromal tumor in the posterior wall of the upper gastric body ( Fig. 2a ). After a single laparoscopic port was created, the lesion was endoscopically removed via the incision of the surrounding mucosa, partial submucosal dissection, and seromuscular incision. After the transoral removal of the lesion, the seromuscular layer was closed using the underlap technique as follows: with its tip pointed inward, the needle was first guided into the peritoneal cavity through the full-thickness defect; then, the tip was gently inserted into the seromuscular layer from the external side and advanced through the layer toward the lumen ( Fig. 2b ). This sequence was repeated alternately on both rims of the seromuscular defect from the anal to the oral direction ( Fig. 2c ). To reduce the size of the defect as quickly as possible, the suture was tightened after each stitch. After the seromuscular closure was complete, the mucosal defect was closed using the conventional EHS technique of continuous suturing ( Fig. 2d ). Finally, double-layer suturing of the seromuscular and mucosal layers was completed with nine punctures in 34 minutes and 12 punctures in 32 minutes, respectively. Pathological R0 resection was achieved without any adverse events.

**Fig. 2:**
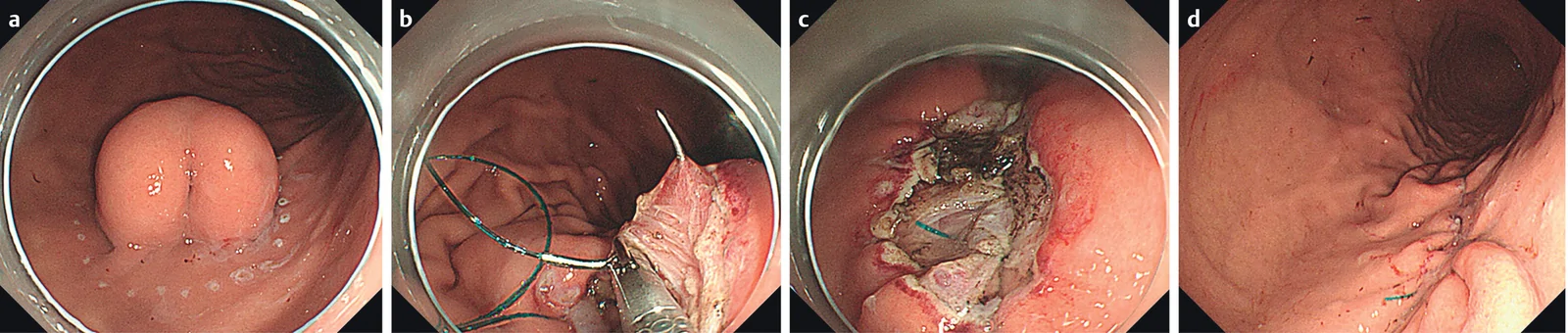
Endoscopic full-thickness resection with the underlap technique of endoscopic hand suturing. ( **a** ) Endoscopic full-thickness resection was performed for the gastrointestinal stromal tumor on the greater curvature of the upper gastric body. ( **b** ) The seromuscular defect was closed with underlap suturing, in which each passage of the needle was directed from the peritoneal to the luminal side. ( **c** ) After the seromuscular closure, the mucosal layer was closed with continuous suturing. ( **d** ). Complete closure of the full-thickness defect was achieved.

The underlap suturing technique was applied successfully to close a full-thickness defect in EFTR; piercing the seromuscular layer with each passage of the needle from the peritoneal to the luminal side enables safe and reliable closure while minimizing the risk of injury to adjacent organs.

Endoscopy_UCTN_Code_TTT_1AO_2AI

## Declaration of GenAI Use

The authors declare that no generative artificial Intelligence was used in the preparation of this manuscript.
